# Coexistence of Lung Cancer and Neurocysticercosis: A Rare Case

**DOI:** 10.7759/cureus.58456

**Published:** 2024-04-17

**Authors:** Julie Nguyen, Ajithkumar Puthillath

**Affiliations:** 1 Internal Medicine, St. Joseph's Medical Center, Stockton, USA; 2 Internal Medicine/Oncology, St. Joseph's Medical Center, Stockton, USA

**Keywords:** metastatic non-small cell lung cancer, multidisciplinary treatments, parasitic disease, neurocysticercosis, adenocarcinoma of the lung

## Abstract

Lung cancer with brain metastasis has a high morbidity and mortality worldwide. Neurocysticercosis is a parasitic infection commonly found in regions with poor sanitation. We present a case with the coexistence of lung cancer and neurocysticercosis. A 57-year-old Caucasian female, with a history of secondhand smoke exposure, presented with a cough. Further evaluation revealed a lesion in the right upper lobe of the lung on a CT scan, a frontal lobe lesion on brain MRI, and hypermetabolic lymph nodes on a PET scan. Biopsies confirmed invasive moderately differentiated adenocarcinoma, indicating stage 4 lung cancer with a solitary brain metastasis. The patient underwent stereotactic radiosurgery for the brain lesion and subsequently received chemoradiation therapy. Upon completion of therapy, the patient showed improvement in both lung and brain lesions. Durvalumab maintenance therapy was initiated. However, a follow-up MRI of the brain revealed a new lesion in the right lateral ventricle. Stereotactic radiosurgery was performed to target this lesion. Five months later, a repeat MRI showed growth of the brain lesion. Given the atypical image finding, a biopsy of the right lateral ventricle lesion was performed, revealing an unexpected diagnosis of calcified parenchymal neurocysticercosis. The patient was referred to an infectious disease specialist who started the patient on dexamethasone without antiparasitic treatment. The co-occurrence of metastatic lung cancer to the brain and neurocysticercosis presents significant diagnostic and therapeutic complexities. Despite stereotactic radiosurgery, the patient's neurologic symptoms failed to improve, and subsequent radiographic assessments yielded inconclusive results. Consequently, a brain biopsy was performed, deviating from the usual practice in cancer management, revealing the unexpected presence of neurocysticercosis. This unforeseen diagnosis underscores the critical significance of contemplating alternative etiologies in patients exhibiting atypical clinical manifestations, particularly in regions devoid of prevalent parasitic infections. This case highlights the challenges in identifying and managing complex cases involving lung cancer and neurocysticercosis, where treatment decisions must balance the need for oncologic control and the management of parasitic infection.

## Introduction

Lung cancer is a common malignancy associated with high morbidity and mortality worldwide. Brain metastasis is a well-known complication of lung cancer [1]. Taenia solium (T. solium), known as the pork tapeworm, is a parasitic flatworm that infects humans and pigs. It is commonly found in regions with poor sanitation and inadequate hygiene practices. In humans, ingestion of T. solium eggs can lead to cysticercosis, a condition characterized by the formation of cysticerci in various tissues, including the muscles, eyes, skin, and brain. Neurocysticercosis, which is a T. solium infection in the brain and central nervous system, may have multiple manifestations, ranging from headache to epilepsy or hydrocephalus [2,3]. Diagnosis of neurocysticercosis is typically with neuroimaging such as a magnetic resonance image (MRI) or computed tomography (CT) scan of the brain, which would reveal cystic lesions, or with enzyme-linked immunosorbent assay (ELISA) tests that can detect specific antibodies against T. solium. Management may involve antiparasitic drugs such as albendazole or praziquantel, corticosteroids such as dexamethasone, and antiepileptic medications if seizures occur [2]. We present a case illustrating the simultaneous occurrence of lung cancer and neurocysticercosis in North America, underscoring the importance of considering atypical diagnoses in patients with lung cancer.

## Case presentation

A 57-year-old Caucasian female, with no significant past medical history, except for years of secondhand smoke exposure, presented with an occasional cough. Further evaluation revealed a T1c and N2 lesion in the right upper lobe on a CT scan. A staging positron emission tomography (PET) scan showed a hypermetabolic spiculated lesion in the right upper lobe, as well as hypermetabolic lymph nodes in the perivascular, paratracheal, and right hilar regions. A right upper lobe CT-guided core biopsy confirmed invasive moderately differentiated adenocarcinoma of pulmonary origin. A mediastinal biopsy also confirmed adenocarcinoma. A screening MRI of the brain revealed a 4 mm solitary frontal lobe lesion, initially suspected to be brain metastasis.

The patient underwent stereotactic radiosurgery for the frontal lobe lesion, followed by chemoradiation therapy with two chemotherapeutic agents: carboplatin and paclitaxel for two cycles. During treatment, she experienced various adverse effects, including leukopenia, anemia, grade 2 mucositis, grade 2 neuropathy, fatigue, and nausea. Subsequent imaging studies showed stable disease in the right basal ganglia, without evidence of new enhancing lesions, and a decrease in the size of mediastinal and right hilar adenopathy. The patient was then started on maintenance immunotherapy with durvalumab.

**Figure 1:**
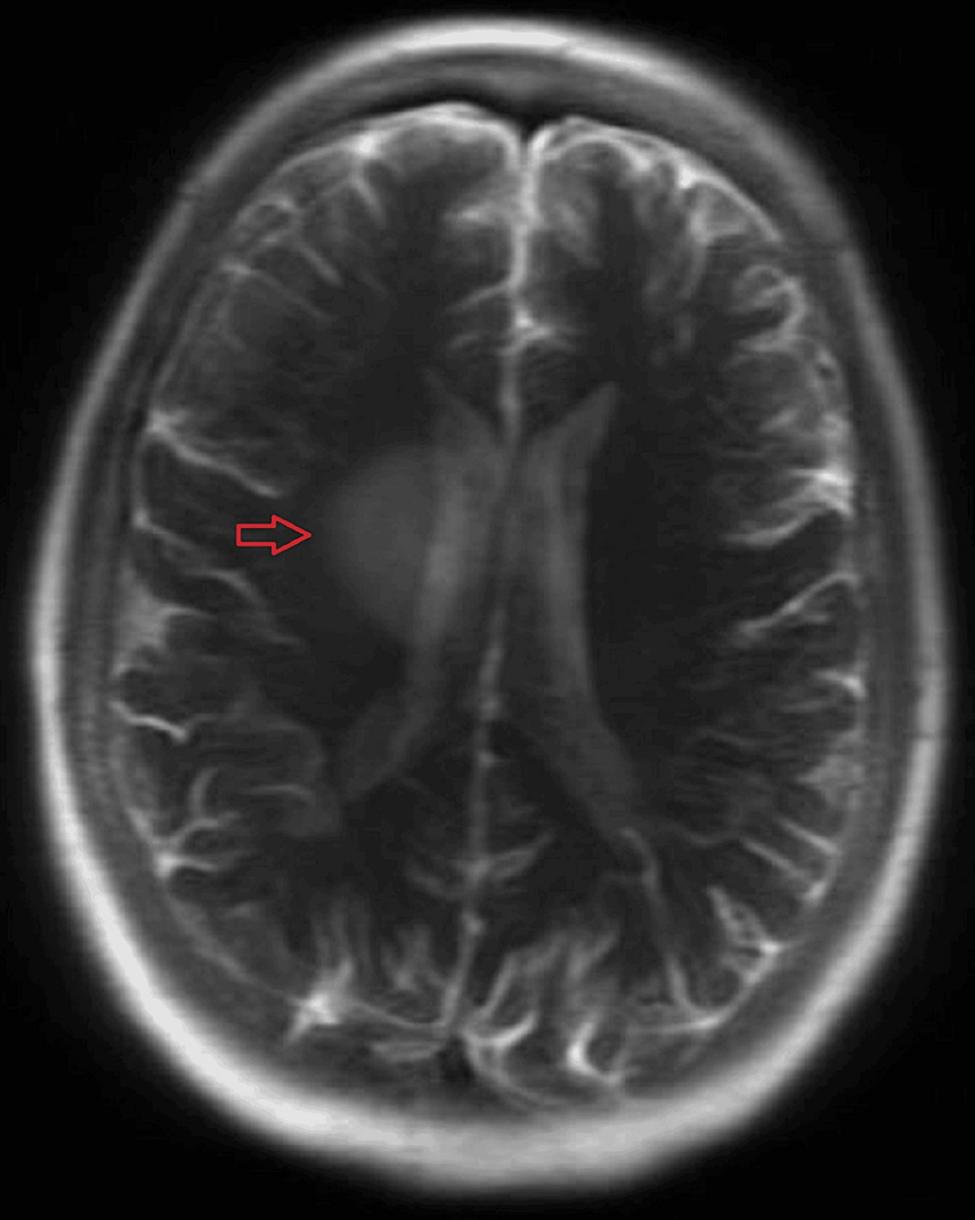
A 2.3 cm rim-enhancing right lateral ventricle periventricular lesion with moderate perilesional edema seen on MRI of the brain Magnetic resonance imaging (MRI)

Upon completion of one year of durvalumab, a PET/CT scan of the lung revealed improvement in the right lung nodule, and an MRI of the brain did not show any masses. However, the patient complained of a constant headache. A subsequent MRI of the brain revealed a 1.1 cm right centrum semiovale metastatic lesion abutting the body of the right lateral ventricle. Stereotactic radiosurgery was performed to target the right lateral ventricle periventricular lesion. Five months later, a repeat MRI showed persistent disease with a 2.3 cm rim-enhancing right lateral ventricle periventricular lesion, as seen in Figure 1. Given the atypical image finding, a biopsy of the right lateral ventricle lesion was performed, revealing an unexpected diagnosis of calcified parenchymal neurocysticercosis. The patient was referred to an infectious disease specialist who started the patient on dexamethasone without antiparasitic treatment.

## Discussion

The coexistence of lung cancer and neurocysticercosis in this patient poses several diagnostic and therapeutic challenges. Lung cancer commonly metastasizes to the brain [1], and the patient's clinical presentation and imaging findings were consistent with brain metastasis. However, the unexpected diagnosis of neurocysticercosis highlights the importance of considering alternative etiologies in patients with atypical presentations, especially in regions where parasitic infections are not prevalent.

In this instance, the patient's initial diagnosis of stage 4 lung adenocarcinoma with solitary brain metastasis prompted an appropriate treatment strategy involving stereotactic radiosurgery and chemotherapy [4]. Following the treatment of her brain metastasis, the patient underwent durvalumab maintenance therapy for a year in accordance with the PACIFIC trial guidelines [5]. However, one year after completing immunotherapy, she developed a persistent headache, leading to an MRI of the brain that disclosed a 1.1 cm lesion in the right lateral ventricle. Stereotactic radiosurgery, a technique delivering concentrated radiation to a targeted region, functioned as an independent treatment for this suspected metastatic brain lesion measuring less than 4 cm [6]. Despite completing radiation therapy, her symptoms persisted. An MRI of the brain revealed a larger lesion, an unexpected occurrence given the patient's adherence to radiotherapy.

The decision to perform a biopsy on brain lesions in lung cancer patients is not standardized and relies on various factors, such as lesion characteristics, neurological symptoms, overall health status, and treatment planning [7]. Brain lesions devoid of atypical features or neurological symptoms may forego a biopsy, and treatment determinations are predominantly based on imaging characteristics [6,7]. However, in this specific case, the patient's persistent headache coupled with the detection of a growing lesion prompted the decision to conduct a biopsy, ultimately disclosing a diagnosis of neurocysticercosis.

The pathophysiology of perilesional edema from a calcified granuloma remains unclear. More recent studies suggest that vesicular cysts evade the host’s inflammatory response, and the scolex enters the cerebrospinal fluid [8]. Usually, the patient is asymptomatic at this stage. Eventually, either the immune response detects the parasite or antiparasitic therapy is started, leading to degeneration of the cyst and resulting in perilesional edema. Patients may experience headaches and seizures during this stage. The cyst then transforms into a nodular lesion that may result in a calcified granuloma [9].

The management of this complex dual diagnosis required collaboration between the oncology, infectious disease, and neurology teams. The initiation of dexamethasone, as recommended by the infectious disease specialist, aimed to control the inflammatory response of neurocysticercosis. Antiparasitic therapy was not warranted as calcified lesions do not contain viable cysts. Symptomatic therapy with antiepileptic drugs for seizures and surgery for hydrocephalus was also recommended [10]; however, the patient did not experience these findings.

The patient’s initial assessment took place at a medical facility within a community setting. Subsequently, her case underwent thorough discussion by the tumor board. Given the intricate nature of her condition, the decision was made to transfer the patient to a specialized tertiary center for more comprehensive evaluation and treatment. The patient’s most recent MRI of the brain after completing immunotherapy and steroids revealed no signs of advancement in the brain lesion. However, the patient still intermittently suffers from migraines.

## Conclusions

This case highlights the challenges in managing complex cases involving lung cancer and neurocysticercosis, where treatment decisions must balance the need for oncologic control and the management of parasitic infection. Further research is necessary to guide the sequencing and coordination of lung cancer treatment and antiparasitic therapy, as well as to explore potential interactions and complications. Increased awareness among healthcare providers regarding the possibility of dual diagnoses is crucial, particularly in regions with a high prevalence of parasitic infections, to ensure timely and appropriate management for patients presenting with complex comorbidities.
